# Pathogenesis of Nonalcoholic Steatohepatitis: Interactions between Liver Parenchymal and Nonparenchymal Cells

**DOI:** 10.1155/2016/5170402

**Published:** 2016-10-16

**Authors:** Nancy Magee, An Zou, Yuxia Zhang

**Affiliations:** Department of Pharmacology, Toxicology & Therapeutics, University of Kansas Medical Center, Kansas City, KS 66160, USA

## Abstract

Nonalcoholic fatty liver disease (NAFLD) is the most common type of chronic liver disease in the Western countries, affecting up to 25% of the general population and becoming a major health concern in both adults and children. NAFLD encompasses the entire spectrum of fatty liver disease in individuals without significant alcohol consumption, ranging from nonalcoholic fatty liver (NAFL) to nonalcoholic steatohepatitis (NASH) and cirrhosis. NASH is a manifestation of the metabolic syndrome and hepatic disorders with the presence of steatosis, hepatocyte injury (ballooning), inflammation, and, in some patients, progressive fibrosis leading to cirrhosis. The pathogenesis of NASH is a complex process and implicates cell interactions between liver parenchymal and nonparenchymal cells as well as crosstalk between various immune cell populations in liver. Lipotoxicity appears to be the central driver of hepatic cellular injury via oxidative stress and endoplasmic reticulum (ER) stress. This review focuses on the contributions of hepatocytes and nonparenchymal cells to NASH, assessing their potential applications to the development of novel therapeutic agents. Currently, there are limited pharmacological treatments for NASH; therefore, an increased understanding of NASH pathogenesis is pertinent to improve disease interventions in the future.

## 1. Introduction

Nonalcoholic fatty liver disease (NAFLD) is the most common type of chronic liver disease in the Western countries and becoming a major health concern in both adults and, tragically, children [1, 2]. The most recent study found the global prevalence of NAFLD was 25% [3]. Individuals with components of metabolic syndrome (MS), such as obesity, insulin resistance, and hyperlipidemia, have an increased risk of developing NAFLD, as positive correlations have been noticed between NAFLD and components of MS [2, 4–6]. NAFLD is closely related to obesity; however, 5–8% of nonobese (lean) subjects also develop NAFLD [7]. One earlier study found that lean-NAFLD has its own metabolic characteristics such as lower fasting glucose and less advanced necro-inflammatory activity and fibrosis compared to obese-NAFLD [8]. A recent study aimed at characterizing lean Caucasian subjects with NAFLD revealed that lean-NAFLD subjects have impaired glucose tolerance and low adiponectin concentrations with an increased rate of mutant patatin-like phospholipase domain-containing 3 (PNPLA3) CG/GG variant compared to lean controls [7]. Another study found Chinese lean-NAFLD is more strongly associated with diabetes, hypertension, and MS than overweight-obese-NAFLD [9].

Encompassing the entire spectrum of fatty liver disease in individuals without significant alcohol consumption, NAFLD is further histologically categorized into nonalcoholic fatty liver (NAFL; steatosis without hepatocellular injury) and nonalcoholic steatohepatitis (NASH) which is characterized by the presence of hepatic steatosis and inflammation with hepatocyte injury (ballooning) with or without fibrosis [10, 11]. NAFL is considered the benign and reversible stage, which arises due to an excessive accumulation of triglycerides in hepatocytes [12]. On the other hand, NASH is a more advanced stage of NAFLD, since the chances of developing more serious diseases such as cirrhosis, hepatocellular carcinoma (HCC), and cardiovascular diseases increase in patients with NASH [13]. A new study showed the mean annual rate of fibrosis progression in NASH is 9%, and NASH overall mortality is 25.6 per 1,000 person-years [3].

Evident from the findings in the aforementioned studies, the pathogenesis of NASH is complex [7–9]. Lipotoxicity-induced oxidative stress and endoplasmic reticulum (ER) stress appear to be the central drivers of hepatic injury in NASH. Recently, additional progress has been made to understand the role of the immune system during NASH progression. For example, inflammation, which occurs in NASH patients and in animal models of human NASH, is induced by various mediators including endotoxins, adipokines, inflammatory cytokines, chemokines, and other inflammatory mediators [14]. The cellular sources of these molecules are broad and include hepatocytes, hepatic stellate cells (HSCs), portal fibroblasts, and immune cells such as neutrophils, macrophages, natural killer (NK) cells, natural killer T (NKT) cells, and lymphocytes [15]. Moreover, what has greatly improved our understanding of NASH is an increasing recognition of importance of interactions between liver parenchymal and nonparenchymal cells as well as crosstalk between various immune cell populations in liver. In this review, we will discuss contributions of hepatocytes and nonparenchymal cells to NASH and assess their potential applications to the development of novel therapeutic agents.

## 2. Hypotheses Describing Pathogenesis of NASH

The pathogenesis of NASH is not yet entirely understood and the mechanism leading to NASH appears multifactorial. A recent retrospective restudy using liver biopsies from patients with NAFL or NASH suggests that rather than being distinct entities NAFL and NASH represent different stages in the progression of NAFLD [16]. Hepatocyte damage is an important factor that drives NAFLD progression. In the initial phase, hepatocyte damage triggers the release of damage-associated molecular pattern molecules (DAMPs) into the microenvironment, which stimulates macrophage activation. This process is influenced by both direct metabolic effects in the liver, such as excessive oxidative stress driven by lipotoxic metabolites, as well as indirect effects coming from the other tissues such as inflammatory initiators released by adipose tissue, the intestine, and the immune system. As a result of these complicated effects, there have been multiple hypotheses describing the pathogenesis of NASH, such as the “two hits,” “three hits,” and “multiple hits” hypotheses.

The “two hits” hypothesis was originally proposed in 1998 [17] in which insulin resistance leads to aberrant lipid accumulation in the liver as the first hit and is followed by a second hit driven by lipotoxic metabolite-induced mitochondrial dysfunction and oxidative stress leading to hepatocyte death and inflammation [18, 19]. In the healthy liver, dead hepatocytes are normally replaced by replication of existing mature hepatocytes; thus normal liver function is maintained. In NASH, however, the replication of mature hepatocytes is inhibited and accompanied by expansion of a progenitor cell population [20]. Those progenitor cells can differentiate either into hepatocyte-like cells or into cholangiocytes, which aid in recovery of normal liver function. However, the abnormal expansion of progenitor cells also contributes to more unfavorable outcomes such as HSC activation and liver fibrosis [21]. Thus, a “third hit,” which drives NASH pathogenesis, involves inadequate hepatocyte proliferation after cell death triggered by insulin resistance induced aberrant lipid accumulation and excessive oxidative stress.

**Figure 1:**
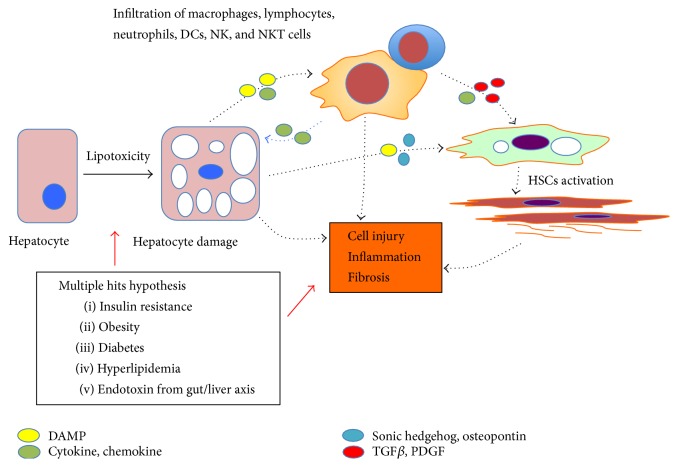
Schematic illustration of NASH pathogenesis. Multiple hits lead to hepatocyte damage involving excessive oxidative stress driven by lipotoxic metabolites. Injured hepatocytes release damage-associated molecular pattern molecules (DAMPs) that initiate an inflammatory response leading to direct recruitment of neutrophils, macrophages, and other components of the innate immune response. Macrophages and damaged hepatocytes, especially ballooned hepatocytes, instigate the release of profibrogenic cytokines and ligands, such as hedgehog and osteopontin. Hepatic stellate cells (HSCs) are subsequently activated and produce excessive extracellular matrix leading to progressive fibrosis. In addition, macrophages promote a proinflammatory microenvironment that initiates an adaptive immune response, likely mediated by T and B lymphocytes.

More recently, a number of different inflammatory mediators released from adipose tissue and the liver/gut axis have been implicated in NASH pathogenesis. Thus, a “multiple hits” hypothesis involving organ-organ interactions in NASH is also appreciated [22]. In this model, NASH pathogenesis is initiated through the triggering of excessive oxidative stress by lipotoxic metabolites. This, in turn, drives hepatocyte death, inflammation, and fibrosis. Additional pathogenic factors from other organs, such as gut-derived endotoxins resulting from increased gut permeability and gut dysbiosis, adipokines secreted from adipose tissue, are all considered crucial to NASH pathogenesis (Figure 1).

## 3. Genetic and Epigenetic Regulation in NASH

It is unknown why some patients have NAFL for many years, whereas others develop the progressive NASH, with or without fibrosis, in only a couple of years. Genetic variation is one important factor that determines whether or not a person has high risk to develop NASH. To date, genome-wide association studies in NAFLD/NASH research identified several genetic variations such as polymorphisms of PNPLA3, transmembrane 6 superfamily member 2 (TM6SF2), farnesyl diphosphate farnesyl transferase 1 (FDFT1), EF-hand calcium binding domain 4B (EFCAB4B), and glucokinase regulator (GCKR), are associated with NASH pathogenesis [23–25]. PNPLA3 gene is located on chromosome 22 and encodes a 481-amino-acid protein that is a triacylglycerol lipase and mediates triacylglycerol hydrolysis in adipocytes. The association of PNPLA3 polymorphism with high risk of NAFLD/NASH has been reported in both adult [26, 27] and pediatric [28] cohorts. A single-nucleotide polymorphism (SNP) in PNPLA3 rs738409 is associated not only with steatosis severity but also with the extent of fibrosis in NASH [29]. An additional study in a hepatoma cell line, Huh7 cells, showed that PNPLA3 rs738409 is associated with reduced enzymatic activity of hydrolyzed, emulsified triglycerides [30].

Epigenetics is an inheritable but reversible phenomenon that affects gene expression without changing the DNA sequence, which includes DNA methylation, histone modifications, and microRNAs [31]. Emerging evidence suggests the importance of the epigenetic machinery coupled with changes of gene expression profile in the regulation of NASH pathogenesis. For example, a genome-wide association study revealed genes involved in cellular apoptosis, lipid biosynthesis, and inflammation response increase during NASH progression, whereas those involved in DNA damage response signal transduction, cholesterol biosynthesis, and carbohydrate metabolism decrease [32]. Another study found that mitochondrially encoded NADH dehydrogenase 6 (MT-ND6) is hypermethylated in human patients with NAFLD and the methylation status is associated with the histological severity of NAFLD [33]. ATP-dependent chromatin remodeling proteins Brahma-related gene 1 (Brg1) and Brahma (Brm) are upregulated in a mouse model of NASH, which induces active histone modifications surrounding the promoters of proinflammatory genes, promoting the basic transcription machinery access to the chromatin and inducing the expression of proinflammatory genes [34]. On the other hand, loss of mitochondrial protein deacetylase SIRT3 causes dysregulation of mitochondrial protein acetylation and accelerates metabolic syndrome and NASH development [35]. In addition, aberrant hepatic expression of microRNAs, such as miR-122, miR-335, miR-29c, miR-34a, miR-155, and miR-200b, has been implicated in the pathogenesis of NASH [36–38].

## 4. Lipotoxic Hepatocyte Injuries, Oxidative Stress, and ER Stress

Lipotoxicity, characterized by excessive free fatty acid (FFA) accumulation within hepatocytes, is known to generate toxic lipid metabolites and cause hepatocyte injury via ballooning and, consequently, initiation of NASH. Ballooned hepatocytes are a cardinal histologic feature of lipotoxic hepatic injury and the magnitude of ballooned hepatocytes correlates with disease severity. In fact, semiquantitation of hepatocyte ballooning is used to calculate the NAFLD activity score (NAS), a measure of disease severity [39], supporting the importance of this phenomenon in disease progression.

Increased dietary intake of FFA, as well as* de novo* lipogenesis and adipose lipolysis, together with impaired FFA oxidation, causes an increase in the flux of FFAs within hepatocytes. Hepatocytes store FFAs as triglycerides. Studies indicate that triglycerides themselves are unlikely to be the cause of hepatocyte injury in NASH. Instead, hepatocyte triglyceride accumulation may act as a protective mechanism to counter FFA-induced lipotoxicity [40]. However, once the threshold of lipid storage is exceeded, the excessive accumulation of FFA leads to production of toxic lipid metabolites, such as ceramides, diacylglycerols, lysophosphatidylcholine, and oxidized cholesterol metabolites [20, 41]. These toxic lipid metabolites promote the overproduction of reactive oxygen species (ROS), which cause liver injury.

Of all the mechanisms related to NASH, oxidative stress has been most widely studied. Oxidative stress is triggered by an imbalance between prooxidants and antioxidants. It is now clear that oxidative stress can mediate liver injury through at least two major mechanisms, direct cell injury and indirect changes of cell signaling pathways. For example, ROS induces activation of nuclear factor *κ*B (NF-*κ*B), a master regulator in the production of proinflammatory cytokines including interleukin-1*β* (IL-1*β*), tumor necrosis factor *α* (TNF*α*), and interleukin-6 (IL-6). Liver-specific inhibition of NF-*κ*B is expected to ameliorate HFD-induced hepatic inflammation [42, 43]. However, the role of NF-*κ*B in NASH pathogenesis is more complicated than people original thought. There is also evidence to suggest that inflammation is required for liver regeneration, which is mediated through antiapoptosis and proproliferative characteristics of NF-*κ*B [44].

FFA-induced oxidative stress also acts as an upstream mechanism to activate ER stress in NASH. ER stress is initiated by conditions associated with protein overload or increased amount of unfolded proteins. Activation of ER stress causes adaptation and recovery of homeostasis; however, severe or prolonged ER stress can ultimately lead to cell death. Recently, attention has turned to the ER due to increasing evidence demonstrating that ER stress is a common feature in NAFLD [45]. For example, one study showed that two ER stress markers, X-box binding proteins (XBP-1) and stanniocalcin 2 (STC2), are increased in human NASH [46]. This study also found that other ER stress proteins, including ATF4, CHOP, and phosphorylated JNK and eIF2*α*, were not significantly changed in NASH samples [46]. Additional studies found activation of ER stress can trigger various inflammatory pathways, such as JNK and NF-*κ*B signaling pathways, further enhancing NASH progression [13, 45, 47]. On the other hand, reduced inflammation ameliorates ER stress-induced liver injury. Kandel-Kfir et al. showed that IL-1*α* deficient mice display reduced inflammation, hepatocyte death, and liver damage in an ER stress-induced steatohepatitis model [48]. These studies help to understand a complex puzzle of NASH pathogenesis, aiding in the elucidation of ER stress risk factors involved in NASH development. Nonetheless, further study is needed and encouraged.

## 5. Inflammatory Mediators and Immune Alterations

Accumulated studies demonstrated that immunological mechanisms, including innate immunity (mediated by neutrophils, macrophages, NK cells, and NKT cells), adaptive immunity (mediated by T and B cells), NLRP3 inflammasome activation, and gut-liver axis, are implicated in the NAFLD progression [49, 50]. As evidence, portal inflammatory infiltrates in human NASH patients are characterized by both broad leukocyte subset markers (CD68, CD3, CD8, CD4, CD20, and neutrophil elastase) and selected inflammatory markers (matrix metalloproteinase 9 and interleukin- [IL-] 17) [51]. The balance of the various immune cell populations and their products involved in inflammatory signaling pathways is crucial to determine NASH attenuation or progression [52].

### 5.1. Macrophages and Gut Microbiota

Macrophages, also termed mononuclear phagocytes, represent a major cell type of innate immunity. Hepatic macrophages consist of resident macrophages called Kupffer cells (KCs) and macrophages that arise from infiltrated bone marrow-derived monocytes. KCs are named after their discoverer, Carl Wilhelm von Kupffer, who originally identified the cells as “sternzellen” or “star cells,” now known to be HSC, but later were correctly identified as macrophages by scientist Tadeusz Browicz [53]. KCs, along with dendritic, NK, and NKT cells, are located in the sinusoidal space of the liver. Given that KCs are the body's primary line of defense against microorganisms that would cause an immune response, this location is optimal for the KCs to carry out their functions in liver. During liver injury, KCs are important in the initial response by rapidly producing cytokines and chemokines, which induces the recruitment of other immune cells, including monocytes, into the liver. Both the infiltrating macrophages and the resident KCs produce proinflammatory and anti-inflammatory cytokines, contributing to the chronic inflammation such as that seen in alcoholic liver disease, NAFLD, and other pathological conditions affecting liver [54, 55].

The liver is constantly exposed to antigens and low levels of endotoxins from the gut as 70% of the liver's blood is supplied from the portal vein. In normal conditions, small amounts of endotoxins from the gut bacteria enter the liver and most of them are eliminated by KCs. Thus, the resident KCs play a critical role in maintaining liver homeostasis and immunological tolerance in the liver. However, the altered composition of microbiota, increase of gut permeability, and hyperresponsibility of KCs to the gut-derived endotoxin can interrupt this tolerance. Recently, gut microbiota analysis revealed that individuals with NAFLD have a lower percentage of* Bacteroidetes* with higher levels of Prevotella and Porphyromonas species compared to healthy controls [56]. Another study found that the inflammasome-mediated dysbiosis of gut microbiota exacerbates hepatic steatosis and inflammation through enhancing liver TNF*α* production [57]. The prolonged exposure to ethanol is known to promote hepatic macrophage hypersensitivity to LPS from the gut and induce a high production of TNF*α*, leading to alcoholic liver disease [58]. Interestingly, patients with NASH harbor modified microbiota that produce endogenous ethanol, suggesting a role for alcohol-producing microbiota in the pathogenesis of NASH [59].

The contribution of macrophages to NAFLD progression is a late outcome of steatosis but an early participant in NASH development, although altered macrophage function has been documented in many stages of NAFLD [60]. Macrophages are extraordinarily versatile cells and exhibit various phenotypes ranging from a proinflammatory classical M1 type to an anti-inflammatory alternative M2 type, depending on the conditions of local microenvironment [61]. The M1 macrophages are abundant in HFD liver and play a critical role in driving inflammation and hepatocyte injury [62]. M2-polarized macrophages counterbalance M1 macrophage-induced inflammation, promoting resolution of inflammation and tissue repair [63]. Favoring M2 macrophages promotes M1 macrophages apoptosis that protects against NAFLD progression [62]. The influence of hepatocyte on macrophages polarization was recently demonstrated in human differentiated macrophage THP-1 cells [64]. In this study, HepG2 cells, a human hepatoblastoma-derived cell line, were pretreated with ER-stress inducers tunicamycin and thapsigargin. The THP-1 cells were then exposed to the conditional medium from HepG2 cells and subsequently displayed M2 phenotype, mediated by the peroxisome proliferator-activated receptor *γ* (PPAR*γ*) signaling pathway. The authors further demonstrated that macrophage M2 activation is initiated by cytokines IL-10 and IL-4 releasing from prolonged ER stressed hepatocytes.

Macrophage-mediated inflammation in NASH is associated with toll-like receptor (TLR) activation; this is particularly true for TLR4 [65]. During liver injury, macrophages release proinflammatory cytokines such as IL-1*β*, TNF*α*, and IL-6 through the activation of TLR4 [66]. When prolonged, this contributes to T cell activation and results in hepatocyte death and subsequent activation of HSCs [67]. Accordingly, TLR4 inhibition or macrophage depletion reduces hepatic damage and prevents NASH development [68, 69].

Interestingly, during NASH, liver macrophages engulf an excessive amount of oxidized low-density lipoprotein (ox-LDL) and form “foam cells” [70]. These macrophage-derived foam cells predominantly contain enlarged lysosomes filled with cholesterol and cholesterol crystals. Additional evidence showed that increased cholesterol storage inside lysosomes of KCs is associated with hepatic inflammation in the context of NASH [71, 72].

Taken together, hepatic macrophages play a critical role in maintaining immune homeostasis of the liver. The important function they play in the pathogenesis of NASH makes them an attractive therapeutic target for NASH treatment. More research on macrophage phenotypes and functions is required to better understand these cells to develop novel macrophage-based therapeutic interventions.

### 5.2. Neutrophils

Neutrophils (also known as neutrophilic granulocytes or polymorphonuclear leukocytes) are the first immune cells to infiltrate the liver after acute injury. Neutrophil infiltration into the liver helps to clear pathogens but may also enhance macrophage cytotoxicity and exacerbate inflammatory state [73]. The contribution of neutrophils in NASH pathogenesis is studied in human NASH and in mouse models. One study found neutrophils infiltrate into the livers of patients with NASH and frequently surround steatotic hepatocytes, resembling the crown-like structures in obese adipose tissue [74]. Moreover, the neutrophil-to-lymphocyte ratio is higher in patients with advanced fibrosis [75]. Transgenic mice expressing HNP-1, a human neutrophil peptide, display enhanced hepatic fibrosis through inducing HSCs proliferation in a choline-deficient and L-amino acid-defined diet-induced mouse model of NASH [76]. In contrast, deletion of elastase, a protease secreted by neutrophils in HFD-induced obese mice, improves liver tissue inflammation with a lower infiltration of neutrophils and macrophages [77]. Beyond this, a better understanding of neutrophil function in the pathophysiology of NASH is still needed and requires further study.

### 5.3. T and B Lymphocytes

T and B lymphocytes mediate the adaptive immune response. For instance, T helper cells, a subgroup of T lymphocytes, are able to drive the activation of the other immune cells. They accomplish this, for example, by helping B cells switch antibody classes, by activating cytotoxic T cells, and by maximizing macrophage phagocytosis through cytokine release [78]. Depending on the cytokine environment, T helper cells can assume a proinflammatory phenotype (Th1), characterized by the release of INF-*γ* and transforming growth factor-*β* (TGF-*β*) or an anti-inflammatory phenotype (Th2), characterized by the release of IL-4, IL-5, and IL-10 [79]. The balance between Th1 and Th2 T cells is important to maintain immune system homeostasis. For example, Th1 and Th2 enhancement can affect macrophage polarization; in particular, Th1 induces macrophages M1 polarization via the release of INF-*γ* [79].

The involvement of adaptive immunity in stimulating adipose tissue inflammation has been extensively studied in obesity. In the initial phase, the fat-resident macrophages secrete chemokines, which recruit CD4+/CD8+ T lymphocytes and NKT cells to the adipose tissue, which, in turn, enhance macrophage activation and proinflammatory mediator release [80]. A very similar mechanism is involved in the initiation of inflammation in NASH pathogenesis, where studies showed that both macrophages and lymphocytes represent the most frequent inflammatory infiltrates of NASH liver [81].

The distinct role of different T cell populations in the pathogenesis of NASH has been recently appreciated. For instance, in human NASH liver biopsy sections, the portal tract infiltrates are dominated by CD8 (+) lymphocytes [51]. Limiting CD8 (+) T-cell expansion by dendritic cells protects mouse liver from NASH development [82]. Th17 cells, a subtype of T helper cells, facilitate leukocyte recruitment through the secretion of various cytokines including IL-17 (IL-17A, IL-17F), IL-21, IL-22, and TNF*α*. Hepatic Th17 cell infiltration is found in NASH [83]. In addition, IL-17 secretion exacerbates hepatic steatosis and inflammation, whereas IL-17 neutralization attenuates LPS-induced liver injury [83]. Furthermore, IL-17A−/− mice were resistant to the development of steatohepatitis, whereas wild-type mice showed progression from NAFL to NASH via the induction of IL-17 and downstream mediators [84]. A most recent study reports the progression from NAFL to NASH is marked by an increase of ratio of Th17/resting regulatory T cells (Tregs) in peripheral blood and liver [85].

By driving T cell activation and secreting proinflammatory cytokines or chemokines, B cells play a critical role in NASH pathogenesis [86]. Lipid peroxidation products, arisen from phospholipid oxidation, interact with cellular proteins and are one of the sources of neoantigens able to promote an adaptive immune response in NASH [87]. As evidence, 40%–60% of patients with NASH have circulating antibodies against lipid peroxidation-derived antigens such as malonyldialdehyde or 4-hydroxynonenal [88]. Furthermore, the high titers of these antibodies are in parallel with increased risk to develop advanced liver fibrosis [89]. Recently, the contributions of B-cells to obesity, diabetes, and NAFLD are extensively examined using animal models. Winer et al. demonstrated that B-cells rapidly increase in serum and adipose tissue of mice fed a HFD [90]. In this study, B cell-deficient mice (B null) fed HFD display a reduced insulin resistance, and adoptive transfer of B cells or IgG isolated from mice fed HFD into B null mice can reverse that phenotype and induce insulin resistance [90]. B cell-activating factor (BAFF, TNFSF13B) is a cell survival and maturation factor for B cells, and overproduction of BAFF is associated with systemic autoimmune disease [91]. Recently, an increase of serum level of BAFF was identified in human NASH, and the serum BAFF level correlates with B-cell content in liver [92]. In addition, BAFF receptor-deficient mice display improved obesity and insulin resistance induced by HFD but also, unexpectedly, show enhanced hepatic steatosis, which indicates a protective role of BAFF in hepatic steatosis [92, 93]. However, contradictory observations on the B-cell's contribution to insulin resistance and NAFLD have also emerged. Bhattacharjee et al. found that B cell-deficient mice (xid mice) fed high fructose drinking water develop the same level of glucose intolerance and insulin resistance as wild-type mice [94], which suggests that B-cells do not play a role in NAFLD progression. The reason for these contradictory observations could be, in part, due to the differences in B-cell-deficient mutant mouse strains and different diet-induced NAFLD models.

Nevertheless, the involvement of adaptive immunity in the processes driving NASH evolution makes T and B cells as attractive therapeutic targets for NASH prevention and treatment. Further studies are required to better understand the interaction between innate and adaptive immunity in sustaining hepatic inflammation and promoting fibrosis in NASH.

### 5.4. NLRP3 Inflammasome

NLRP3 inflammasome is a large, intracellular multiprotein complex expressed in both parenchymal and nonparenchymal cells of the liver. In response to various cellular danger signals, NLRP3 inflammasomes activate caspase-1 and release mature IL-1*β* and IL-18 [95]. Interestingly, recent studies revealed NLRP3 inflammasome activation as an emerging factor contributes to NASH development. For example, the expressions of NLRP3 components, pro-IL-1*β* and pro-IL-18, are markedly increased in both mouse models and humans with NASH [96, 97]. Moreover, NLRP3 knockout mice or IL-1*α* or IL-1*β* knockout mice are protected from diet-induced liver injury, inflammation, and fibrosis [97, 98]. Another study demonstrates that selective inhibition of caspase-1 alleviates hepatic steatosis, inflammation, and fibrosis in a diet-induced mouse model of NASH [99]. These studies strongly suggest that NLRP3 inflammasome may serve as a potential therapeutic target for the treatment of NASH.

## 6. Role of HSCs in NASH Progression

Liver fibrosis is a condition in which an excessive amount of extracellular matrix (ECM) proteins, like type I collagen, accumulates in the liver. This buildup of ECM occurs in most types of chronic liver diseases including NAFLD [100]. Although many cell types, including the hepatocytes and sinusoidal endothelial cells have been identified as contributors of ECM components, liver myofibroblasts, originally from HSCs (from the word of Latin origin,* stella*, meaning* star*), portal fibroblasts (PFs) or mesothelial cells are the major source of ECM [101]. The role HSCs play in fibrosis is unequivocal. Much data has demonstrated that HSC activation precedes fibrogenesis and that a lack of HSC activation halts the process [102–104]. Lipid accumulation, as that seen in NAFLD, triggers a profibrogenic response from HSCs [12]; therefore an overview of fibrogenesis in NASH is critical to understanding NASH progression.

Although HSCs only make up about 1.4 percent of the liver cell population [105], their effect on overall liver homeostasis, particularly in cases of liver injury, is worthy of attention. HSCs are likely mesenchyme in origin, due to the fact that they produce alpha-smooth muscle actin (*α*-SMA) when activated and express vimentin and desmin [105]. HSCs reside in the space between hepatocytes and the liver sinusoidal endothelial cells, known as the space of Disse [106]. In healthy liver, HSCs exist in a quiescent state, storing vitamin A and lipids, a function, which led to an alternative name for HSC, the lipocyte [106, 107]. Upon liver injury, HSCs become highly proliferative, losing vitamin A and lipid droplets. In the same process, HSCs commence in mass production of a fibrotic extracellular matrix profuse with type I collagen [103] that allows the activated HSCs to be characterized as a myofibroblast-like cell.

For over two decades, researchers have gathered enough convincing data suggesting that HSCs, indeed, are the main cells involved in the production of extracellular matrix (ECM) in liver fibrosis [105, 108]. Other cell types like PFs and smooth muscle cells (SMCs) also contribute to the synthesis of connective tissue proteins as well [103]. For instance, the PFs, but not the HSCs of the hepatic sinusoid, play a predominant role in the early stage of cholestatic fibrosis when portal tracts are injured [103]. HSCs resemble and function in a similar manner as PFs when they are active. However, when quiescent, HSCs and PFs differ functionally as well as with respect to from which embryologic tissue they arise [109]. Different markers exist which can be used to distinguish between HSCs and PF. For example, recent research suggests that HSCs can be accurately distinguished from PFs based in expression of cytoglobin (CYGB): the CYGB protein is found in both quiescent and active HSCs but not in PFs after immunohistochemistry [110]. In addition, HSCs are positive for desmin and PFs are positive for elastin instead [101].

HSC activation involves two phases: the* initiation *phase and the* perpetuation *phase [105]. During the initiation phase, HSCs proliferate and become myofibroblast-like in response to proliferative and fibrogenic cytokines. Only activated HSCs express alpha2-macroglobulin, P100, CD95L, and reelin, which makes these proteins good identifiers for HSC activity [103, 111, 112]. There are many cells involved in activating HSC. For example, hepatocytes, liver sinusoidal endothelial cells, macrophages, NK cells, and lymphocytes play roles in the activation process [113]. Those cells secrete mediators that affect HSC activation. Of the mediators that are released, platelet-derived growth factor (PDGF) and transforming growth factor beta (TGF-*β*) are the two best-described growth factors. PDGF is involved in the signaling process required for HSC proliferation, while TGF-*β* promotes collagen production [114]. The increase of ECM components (fibrillar collagens such as type I collagen) and inhibitors of matrix-degrading enzymes, like tissue inhibitor of matrix metalloproteinases (TIMP), occurs in the second phase of HSC activation—an event resulting in matrix accumulation, especially at sites where many activated HSCs reside [100].

Extensive studies have investigated how HSCs are activated in NAFLD. Lipid metabolites accumulation in hepatocytes induces TGF-*β* signaling and impairs adiponectin activity, supporting a key role for lipotoxicity in the development of hepatic fibrosis [115]. Recent data demonstrate a positive correlation between the Notch signaling pathway and HSC activation. In TGF-*β*-activated HSCs, Notch pathway components are significantly increased and inhibition of Notch signaling decreases HSC activation [116]. Schnabl et al. demonstrated that TGF-*β*-activated kinase 1 (TAK1)/c-Jun N-terminal kinase (JNK) and p38 pathways work collaboratively in HSC activation. TAK1/JNK promotes HSC proliferation while p38 decreases HSC proliferation [117]. Another recent study suggests that osteopontin and high mobility group box 1 (HMGB1) releasing from necrotic hepatocyte also play a key role in HSC activation [118]. Most recently, Dr. Guy and coworkers discovered that ballooned hepatocytes generate sonic hedgehog (Shh), a ligand of the hedgehog-signaling pathway, which promotes HSC activation and drives NASH progression in mice [119]. Those studies support the notion that HSCs shift from a fairly quiescent state to an ECM-producing machine in NASH and the regulation for that process is quite complex.

## 7. Therapeutic Options

There is no pharmacological treatment for NASH. However, therapeutic options exist to manage NASH symptoms such as probiotics for gut dysbiosis, physical activity, and weight loss for obesity and diabetes [120]. Targeting PPARs are of specific interest due to the suspected roles that these nuclear receptors have in preventing hypertriglyceridemia and type 2 diabetes (two risk factors for NAFLD) [121, 122]. Targeting hepatic macrophages is also one of the focus areas for therapeutic options [123]. This is especially true since hepatic macrophages are involved in many processes throughout NAFLD progression. Another suggestion is to target the main cells responsible for hepatic fibrosis, HSCs. Proposed methods include, but are not limited to, targeting TGF-*β*1, PDGF, and PPARs (specifically PPAR*γ*) [124].

As we have discussed, oxidative stress is a key feature of NAFLD progression. Vitamin E is an antioxidant, which prevents oxidative stress associated with JNK activation. In 2010, NIDDK sponsored a PIVENS trial (PPAR*γ* agonist pioglitazone, vitamin E, or placebo for NASH, NCT00063622) in 247 adults with NASH without diabetes. The improvement in histologic features of NASH was assessed with the use of a composite of standardized scores for steatosis, lobular inflammation, hepatocellular ballooning, and fibrosis. It turned out that vitamin E was superior to placebo for the treatment of NASH in adults without diabetes [125]. A later study found that the treatment response in vitamin E group is correlated with the loss of Shh+ hepatocytes and an improvement against Hh-promoted NASH progression [126, 127]. Another promising therapeutic option includes glucagon-like peptide-1- (GLP-1-) based therapies, which promote hepatocyte survival via reduction of hepatic fat accumulation and unfolded-protein response [120, 128].

## 8. Conclusions

Our knowledge of NASH pathogenesis has been greatly advanced through animal models and* in vitro* studies, as well as through the examination of liver specimens from patients with NAFLD. The pathogenesis of NASH and its progression to fibrosis are very complex and occur in response to a chronic inflammatory state in the setting of obesity, insulin resistance, hepatic steatosis, and oxidative stress. In any case, the ability to treat a disease relies heavily on the knowledge of disease etiology. So far, the main treatment options are to relieve or prevent the symptoms of NAFLD via changing diet, weight loss, exercise, or bariatric surgery [129]. Progress in this aspect has greatly improved recently. However, more remains to be uncovered regarding the connections between, and the orders of, the pathways involved in NASH pathogenesis particularly for patients whose liver disease does not respond to these behavioral or surgical options. Additionally, when these proposed treatment options were considered, there was not sufficient data or evidence to show the treatments are effective to ameliorate NASH in human patients [130]. As we have discussed above, the pathogenesis of NASH involves multiple mechanisms that affect both liver parenchymal and nonparenchymal cells; thus a multipronged strategy to design and implement multimodality pharmacologic approaches targeting multiple mechanisms could possibly be more successful than single-agent use. Nonetheless, it is hoped that an increased understanding of NASH pathogenesis and progression, and particularly the mechanism of triggering immune response and liver fibrosis, will provide better targets for therapeutic intervention in this growing common disease.
